# Nuclear adenomatous polyposis coli elevates *STAT1* and reduces *CXCL1,2, and 3* expression and inhibits neutrophil recruitment

**DOI:** 10.1016/j.cellsig.2025.111957

**Published:** 2025-06-20

**Authors:** Alexander Q. Sandoval, Anika James, Kristi L. Neufeld

**Affiliations:** Department of Molecular Biosciences, University of Kansas, Lawrence, KS 66045, USA

**Keywords:** Inflammation, CXCL, Adenomatous polyposis coli (APC), STAT1, Neutrophil, Transcriptional regulation

## Abstract

Adenomatous polyposis coli (*APC*) mutations and chronic inflammation can each promote colon cancer. Though both mice and humans with germline *APC* mutations show reduced tumorigenesis if treated with anti-inflammatory agents, direct links between APC and inflammation remain incomplete. In the current study, we examine a novel role for APC in intestinal inflammation via inhibition of neutrophil-recruiting chemokines CXCL1, 2, and 3. Patients with colorectal adenocarcinoma, the majority of whom would be expected to harbor *APC* mutations, showed upregulated CXCL1, 2, and 3 expression at early stages of disease. APC induction in cultured human colon cells reduced levels of CXCL1 and CXCL2 proteins and *CXCL1*, *CXCL2*, and *CXCL3* RNAs and increased expression of signal transducer and activator of transcription 1 (*STAT1*), a potential negative regulator of *CXCL1* transcription. By mining published Chromatin-immunoprecipitation sequencing (ChIP-Seq) data, we found regions of the *STAT1* promoter and upstream CpG island as APC-bound. Methylation-specific PCR and bisulfite sequencing each revealed decreased methylation of the *STAT1* CpG island upon APC induction. Intestinal tissue explants from mice compromised for nuclear Apc (Apc^mNLS/mNLS^) secreted more CXCL1 and CXCL2 than wild-type explants. Conditioned media from APC-expressing cells recruited fewer neutrophils in a trans-well migration assay. In vivo, colon and ileal tissues from Apc^mNLS/mNLS^ mice displayed more neutrophils than Apc^+/+^ mice. Experimental evidence from in vitro and in vivo systems validates that nuclear APC can inhibit inflammation by suppressing neutrophil-recruiting chemokines CXCL1, 2, and 3, potentially via epigenetic regulation of *STAT1*. These findings offer a new target for managing inflammation in inflammatory bowel disease and reveal a new mechanism by which APC loss enables cancer progression.

## 1. Introduction

Ulcerative colitis (UC) is a chronic inflammatory bowel disease (IBD) with an estimated 5 million cases worldwide in 2023 [1]. UC is characterized by recurring bouts of epithelial wounding and repair in the colon and rectum, resulting in a breached mucosal barrier. The compromised barrier allows subsequent microbial invasion, inflammation, and a dysregulated immune response [1]. Multiple factors contribute to UC susceptibility, including diet, age, and genetics [2]. Since there is no established cure for UC, current treatments are mostly aimed at mitigating chronic and symptomatic inflammation [3]. This mitigation is imperative, as inflammation is a major risk factor in colorectal cancer development. UC patients are two to three times more likely to develop CRC than the general population [4], and the degree of inflammation in IBD patients has been correlated with CRC risk [5–8]. As such, it is critical to identify and characterize novel signaling pathways and proteins that regulate inflammation which can be used to develop new IBD interventions.

Adenomatous Polyposis Coli (APC) mutations are observed at an early stage of development for ~80 % of all colorectal cancers. Both mice and humans with germline APC mutations show reduced tumorigenesis if treated with anti-inflammatory agents [9–12]. Though most recognized as a cytoplasmic antagonist in the Wnt signaling pathway [13], APC has other functions and characteristics, including the ability to localize to the nucleus [14]. Since demonstrating that APC can shuttle between the cytoplasm and nucleus [14,15], we have focused efforts on determining roles for nuclear APC in maintaining intestinal homeostasis. To this end, we generated a mouse model with mutations introduced into Apc nuclear localization signals (NLS), resulting in primarily cytoplasmic Apc [16]. Throughout this paper we will use conventional nomenclature: Apc represents the murine protein; APC represents the human protein. When our Apc^mNLS/mNLS^ mice were treated with colitis-inducing dextran sodium sulfate (DSS), they displayed more severe inflammation, weight loss, colon shortening, visible lymphoid follicles, and edema than treated wild-type mice [17]. Notably, even untreated Apc^mNLS/mNLS^ mice expressed elevated RNA levels of inflammatory mediators *cyclooxygenase-2 (COX-2)* and *macrophage inflammatory protein-2* (*MIP-2)*, the latter also known as C-X-C motif chemokine ligand 2 (CXCL2) [17]. Aside from APC’s transcriptional repression of the Wnt target gene *COX2*, direct links between APC and inflammation remain elusive. Specifically, the mechanism by which nuclear APC inhibits chemokines like CXCL2 and thereby reduces intestinal inflammation is unknown.

Chemokine expression can be controlled by numerous signals, including through transcriptional inhibition by the transcription factor STAT1. Hankey et al. identified 3985 genomic regions enriched by APC Chromatin-immunoprecipitation sequencing (ChIP-Seq) [18]. Mining these sequences, we found that several contained the *STAT1* promoter and upstream CpG island, hinting at a potential direct regulatory mechanism. CXCL1, CXCL2, and CXCL3 are chemokines that bind to CXCR2 receptors on circulating neutrophils, resulting in their recruitment [19]. Neutrophils are important leukocytes of the innate immune system, well-established as first responders of the acute inflammatory response that defends against various pathogens [20]. In the context of IBD, neutrophils have both damaging and protective effects on the intestinal epithelial barrier. Neutrophils can damage the epithelial barrier by releasing high levels of reactive oxygen species, myeloperoxidase, defensins, lysozyme, elastase, and neutrophil extracellular traps [21]. This release can amplify the immune response and damage the mucosa. In contrast, neutrophils are also critical for maintaining a host defense against pathogens, promoting intestinal epithelial wound repair, and resolving inflammation [22].

In the current study, we further investigated APC’s role in intestinal inflammation by focusing on APC regulation of neutrophil-recruiting chemokines CXCL1, CXCL2, and CXCL3. By examining intestinal tissue explants from Apc^mNLS/mNLS^ mice, a human colon cell line inducible for full-length, functional APC, and patient data from TCGA, we obtained evidence that nuclear APC can inhibit inflammation by suppressing neutrophil-recruiting chemokines via *STAT1*.

## 2. Materials and methods

### 2.1. Cell culture

HT29 cells with zinc-inducible APC (HT29-APC) or β-galactosidase (HT29-β-gal), generously provided by Dr. Bert Vogelstein (Johns Hopkins University), were maintained at 37°C and 5 % CO_2_ in McCoy’s 5 A (Iwakata & Grace Mod.) with L-glutamine medium (Corning Inc., Corning, NY, USA) supplemented with 10 % fetal bovine serum (R&D Systems, Minneapolis, MN, USA) and 0.6 mg/mL hygromycin B gold (Invivogen, San Diego, CA, USA). Cells were passaged after reaching 70–80 % confluence and maintained until 20 passages. For all experiments, hygromycin was eliminated from the media. Expression of APC or β-gal in the HT29 cells was induced by adding 150 μM ZnCl_2_ (Thermo Fisher Scientific, Waltham, MA, USA) to the medium for 48 h.

The HL-60 cell line (American Type Culture Collection, Manassas, VA, USA) was maintained at 37 °C and 5 % CO_2_ in RPMI-1640 with l-glutamine medium (Corning Inc.) supplemented with 10 % fetal bovine serum (R&D Systems). HL-60 cells were differentiated into neutrophil-like dHL-60s by exposure to 0.3 μg/mL all-trans-retinoic-acid (Cayman Chemical, Ann Arbor, MI, USA) for five days at which time ~ 35 % appeared to have neutrophil-like nuclear morphology.

### 2.2. Western blot

HT29-APC and HT29-β-gal cells (3 ×10^5^) were seeded in wells of a 6-well plate. The following day, protein expression was induced with 150 μM ZnCl_2_ for 48 h at which time, cells were lysed in 95 °C high-salt sample lysis buffer (30 % 10× PBS, 2 % SDS, 20 % glycerol, 2.5 % β-mercaptoethanol). Cells were scraped using a pipette tip and transferred to Eppendorf tubes. Lysates were heated at 95 °C for 3 min and DNA sheared with 10 passages through insulin syringes with 27G × 1/2″ needles. Lysates were heated for 3 min before separation with 7.5 % SDS-polyacrylamide gel electrophoresis using Tris-Glycine running buffer followed by transfer to a 0.45 μm pore size nitrocellulose membrane (Cytiva, Marlborough, MA, USA). After transfer, the membrane was dried at room temperature for 1 h and then blocked in Intercept TBS Blocking Buffer (LI-COR, Lincoln, NE, USA) for 6 h at 4 °C. The membrane was incubated overnight with anti-APC-M2 rabbit pAb (1:500) [23] and anti-β-actin mouse mAb (1:1000, MilliporeSigma, Burlington, MA, USA) at 4 °C. Membranes were washed 3× with TBS-Tween before incubation with IRDye 680LT donkey anti-mouse secondary Ab and IRDye 800CW goat anti-rabbit secondary Ab (1:15000, LI-COR) for 1 h at room temperature. Three washes were performed again before membranes were visualized using a LI-COR Odyssey CLx imaging system.

### 2.3. Measurement of CXCL1 and CXCL2 secretion from HT29 cells with enzyme linked immunosorbent assays (ELISA)

HT29-APC and HT29-β-gal cells (3 × 10^5^) were seeded in each well of a 6-well plate. The following day, protein expression was induced with 150 μM ZnCl_2_. After 24 h, cell media was treated with lipopolysaccharide (LPS, 20 ng/mL, Invitrogen, Waltham, MA, USA) to stimulate cytokine activity. After 24 h, “conditioned” media were collected. The Human CXCL1/GROα DuoSet ELISA Kit and the Human CXCL2/GROβ DuoSet ELISA Kit (Bio-Techne, Minneapolis, MN, USA) were used as per manufacturer’s instructions to assess the level of CXCL1 and CXCL2 into the media. Bio-Techne’s DuoSet Ancillary Reagent Kit 2 was required for both kits. Due to differing growth rates of HT29-APC cells and HT29-β-gal cells (Supp. Fig. 1), the number of cells in each well was determined using a hemocytometer, and the amount of CXCL1 or CXCL2 was standardized to cell count.

### 2.4. RNA extraction and quantitative real-time polymerase chain reaction (qRT-PCR) and human tissue gene expression analysis

HT29-APC and HT29-β-gal cells were plated, exposed to ZnCl_2_, and treated with LPS as for ELISA experiments. Cells were lysed with TRIzol Reagent (Invitrogen) and RNA extracted using phenol-chloroform. cDNA was formed by incubating 1 μg RNA, Random Primer 6, 10 mM dNTPs, and M-MuLV Reverse Transcriptase (New England Biolabs, Ipswich, MA, USA) for 1 h at 42 °C. qRT-PCR was performed using a QuantStudio3 Real-Time PCR System (Thermo Fisher Scientific) and PowerUp SYBR Green Master Mix (Thermo Fisher Scientific). Primer sequences are shown in Table 1 [24,25]. Each reaction was performed in triplicate and the experiment was repeated three times. Cycle settings: initial 2 min @ 50°C, 2 min @ 95°C, followed by 40 cycles of 1 s @ 95°C, 15 s @ 58.1°C, and 1 min @ 72°C. The relative mRNA, normalized to *GAPDH*, was determined using 2^−ΔΔCt^ [26]. Primer efficiencies were optimized and differences in primer efficiencies were accounted for using the Pfaffl Eq. [27].

*CXCL1–3* RNA levels were assessed in human tissue samples using the GEPIA2 tool [28]. The TCGA data analyzed included colon (275 tumor and 349 normal) and rectal (92 tumor and 318 normal) samples and compared tumor expression in Transcripts Per Kilobase Million (TPM) with matched TCGA normal and GTEX data. For tumor stage analysis, the jitter size was set to 0.4 and the [Log2FC] cutoff to 1.

### 2.5. Methylation-specific PCR

HT29-APC and HT29-β-Gal cells (4 ×10^5^) were plated in each well of a 6-well plate and treated with ZnCl_2_ the following day. Two days following zinc treatment, gDNA was harvested using a DNeasy Tissue and Blood Kit (QIAGEN) as instructed. gDNA (350 ng) underwent bisulfite conversion using an EZ DNA Methylation Kit (Zymo Research, Irvine, CA, USA) as instructed. Bisulfite-converted DNA was PCR amplified using GoTaq Flexi DNA polymerase and primers designed to anneal with methylated (M) or unmethylated (U) regions of the *STAT1* promoter CpG island. Primers were designed using MethPrimer software and are listed in Table 2 [29]. PCR was performed using a T100 Thermal Cycler (Bio-Rad) with the following cycle settings: 2 min @ 95°C, followed by 34 cycles of 30 s @ 95°C, 30 s @ 50°C, 1 min @ 72°C with a final 5 min extension at 72°C. PCR products were separated on a 2 % agarose gel and imaged using a FluorChem E Imaging System (Biotechne). M primers were expected to produce a 175 bp product, and U primers, a 182 bp product. As a positive control for M primers, 20 μL reactions containing 1 μg of HT29-APC or HT29-β-gal gDNA were treated with CpG methylase according to manufacturer’s standard reaction setup (Zymo Research). Reactions were incubated at 30 °C for 8 h with an extra 1 μL of CpG methylase added at 2 h. Samples were then incubated at 65 °C for 20 min prior to bisulfite conversion and methylation-specific PCR as described.

### 2.6. Bisulfite sequencing

The *STAT1* CpG island of bisulfite-treated HT29-APC and HT29-β-Gal gDNA was PCR amplified using GoTaq DNA Polymerase (Promega) and primers in Table 3. Cycle settings were the same as for methylation-specific PCR, except using a 53°C annealing temperature. PCR products were separated on a 1.5 % gel and purified using QIAquick Gel Extraction Kit (QIAGEN). PCR products were sequenced by Sanger method by Genewiz (Azenta Life Sciences, Burlington, MA, USA). Sequence chromatogram data was analyzed using SnapGene (GSL Biotech LLC, San Diego, CA, USA) software to determine peak heights of cytosine and thymine at each CG site within the CpG island. For each CG site, the percent methylation was calculated as the ratio of the cytosine peak height and the total peak heights of cytosine and thymine [C/(C + T)].

### 2.7. MethMarkerDB database analysis

The MethMarkerDB database [30], which integrates 658 whole-genome bisulfite sequencing data sets, was used to examine the methylation status of the *STAT1* CpG island in normal colons and colorectal cancers of humans. Methylation levels for the *STAT1* CpG island, chr2:191020117–191,020,584 (hg 38) were determined in colorectal cancer and normal samples. One data point from each sample was more than two standard deviations away from the sample mean, classified as an outlier, and omitted from the graph.

### 2.8. Transwell migration assay

HL-60 cells were differentiated into neutrophil-like cells (dHL-60) by incubating with all-trans-retinoic-acid (0.3 μg/mL) for 5 days. HT29-APC and HT29-β-Gal cells (2 × 10^5^) were plated in each well of a 24-well plate and exposed to 150 μM ZnCl_2_ the following day. Conditioned media from the HT29 cells were collected 48 h later, and the HT29 cells in each well were counted using a hemocytometer. Transwell inserts (3 μm pore, cellQART, Northeim, Germany) were placed in a separate 24-well plate. The prepared dHL-60s were diluted to a concentration of 2 × 10^5^ dHL-60/mL and 200 μL of the diluted dHL-60 solution was placed in the top chamber of each transwell insert. The cells were allowed to settle for 10 min at 37 °C and 5 % CO_2_ before 600 μL of the HT29-APC or HT29-β-Gal conditioned media was placed in the well beneath the transwell insert. Following six hours incubation at 37 °C and 5 % CO_2_, the media from the bottom chamber of the transwell insert was collected, and dHL-60 cells were counted in this media. Migrated dHL-60 cell counts were standardized to HT29 cell counts.

### 2.9. Quantification of neutrophils in Apc^+/+^ and Apc^mNLS/mNLS^ mouse colons and ileal tissue

Colons of Apc^+/+^ and Apc^mNLS/mNLS^ mice (*n* = 6 for each) treated with 5 % DSS for one week to induce inflammation were Swiss-rolled, paraffin-embedded, sectioned, and stained with hematoxylin and eosin as previously described [17]. Using a Keyence Microscope (40× magnification, Keyence Corporation of America, Itasca, IL, USA), three images were taken for each region of the colon (proximal, mid, and distal) for each mouse. Neutrophils were identified based on their multi-lobed nuclear morphology and counted in proximal, mid, and distal colon lamina propria sections. Neutrophil counts were standardized to the area as calculated using ImageJ. For untreated Apc^+/+^ and Apc^mNLS/mNLS^ mice (*n* = 4 and 5, respectively), ileal tissue was removed, and the distal 3 cm used for explant culture. The remaining ilea were processed as described [31] and stained for neutrophil marker Ly6G. Briefly, following antigen retrieval in citrate buffer (pH 6.2, 0.05 % Tween-20) at 95 °C for 40 min, sections were blocked using Vector^®^ M.O.M. Mouse IgG Blocking Reagent and then Protein Diluent (Vector Laboratories, Cat. #BMK-2202) as instructed by manufacturer. Tissue was incubated overnight at 4 °C with anti-Ly6G (R&D Systems, Cat. #MAB1037) at 3 μg/mL in M.O.M. diluent. After three PBS washes, sections were treated with biotinylated anti-mouse IgG (Vector^®^ M.O.M. kit) for 30 min, washed, and incubated with HISTOSTAIN^®^-PLUS Streptavidin–HRP (Invitrogen, Cat. #50–420Z) for 20 min. Signal was developed in DAB-Plus substrate (Invitrogen, Cat. #00–2020) for up to 5 min and enhanced with DAB enhancer (1 min), counterstained with Hematoxylin solution according to Mayer (Sigma Aldrich Cat. # 51275) for 1 min, dehydrated through ethanol, cleared in xylene, and mounted with EcoMount (Biocare Medical Catalog# EM897L). Neutrophils were identified by their distinct morphology and Ly6G positivity. For both colon and ileum, neutrophils were scored in 3 images from each of 3 tissue sections for each mouse and normalized to area analyzed.

### 2.10. Ethical approval

All authors had access to the study data and had reviewed and approved the final manuscript. This research complied with all relevant federal guidelines and institutional policies. The University of Kansas Institutional Animal Care and Use Committee approved all procedures that used animals, and experiments were conducted according to the Guide for the Care and Use of Laboratory Animals (National Research Council, 2011). C57BL/6 J mice were maintained at the animal care unit at the University of Kansas according to animal use statement number 137–01. The animals were housed in standard ACU conditions (temperature of 23 °C, with room lights on from 06.00 to 18.00 h), were provided with water, fed ad libitum with Teklad Global 18 % Protein Rodent Diet (Envigo, Indianapolis, IN, USA, no. 2918), and housed in cages in the same room (with cages housing a maximum of five mice). The investigators understand the ethical principles under which the journal operates, and their work complies with its animal ethics checklist.

### 2.11. Mouse explant culture

The explant protocol was adapted from Dr. Livie Chatelais’s protocol [32]. Mice were humanely killed and their small intestines harvested. For each mouse, distal ileum was opened and briefly rinsed with PBS then three pieces, each about 1 cm, were excised, weighed, and placed in a 24-well plate containing RPMI-1640 media supplemented with 1 % pen/strep. The explants were incubated at 37 °C and 5 % CO_2_ for 24 h. Following incubation, the media were collected, centrifuged, snap frozen, and stored at −80 °C until analysis. CXCL1 and CXCL2 levels in the explant media were determined using a Mouse CXCL1/KC DuoSet ELISA Kit and Mouse CXCL2/MIP-2 Quantikine ELISA Kit (Biotechne) as instructed. CXCL1 and CXCL2 amounts were standardized to explant weights. Triplicate values for each sample were averaged. A few explants did not contain measurable CXCL1 or CXCL2 and were excluded from the data.

### 2.12. Statistical tests used to analyze data

An unpaired Student’s *t*-test was used to calculate statistical significance for the following: secreted protein levels (ELISA), gene expression (RT-PCR), bisulfite sequencing chromatogram peaks, MethMarker DB results, neutrophil migration and levels in tissue. Statistical significance for gene expression in human patient samples was calculated using one-way ANOVA.

## 3. Results

### 3.1. APC induction in human colon cancer cells leads to decreased levels of CXCL1, 2, and 3 mRNA and secreted CXCL1 and CXCL2 protein

**Fig. 1.**
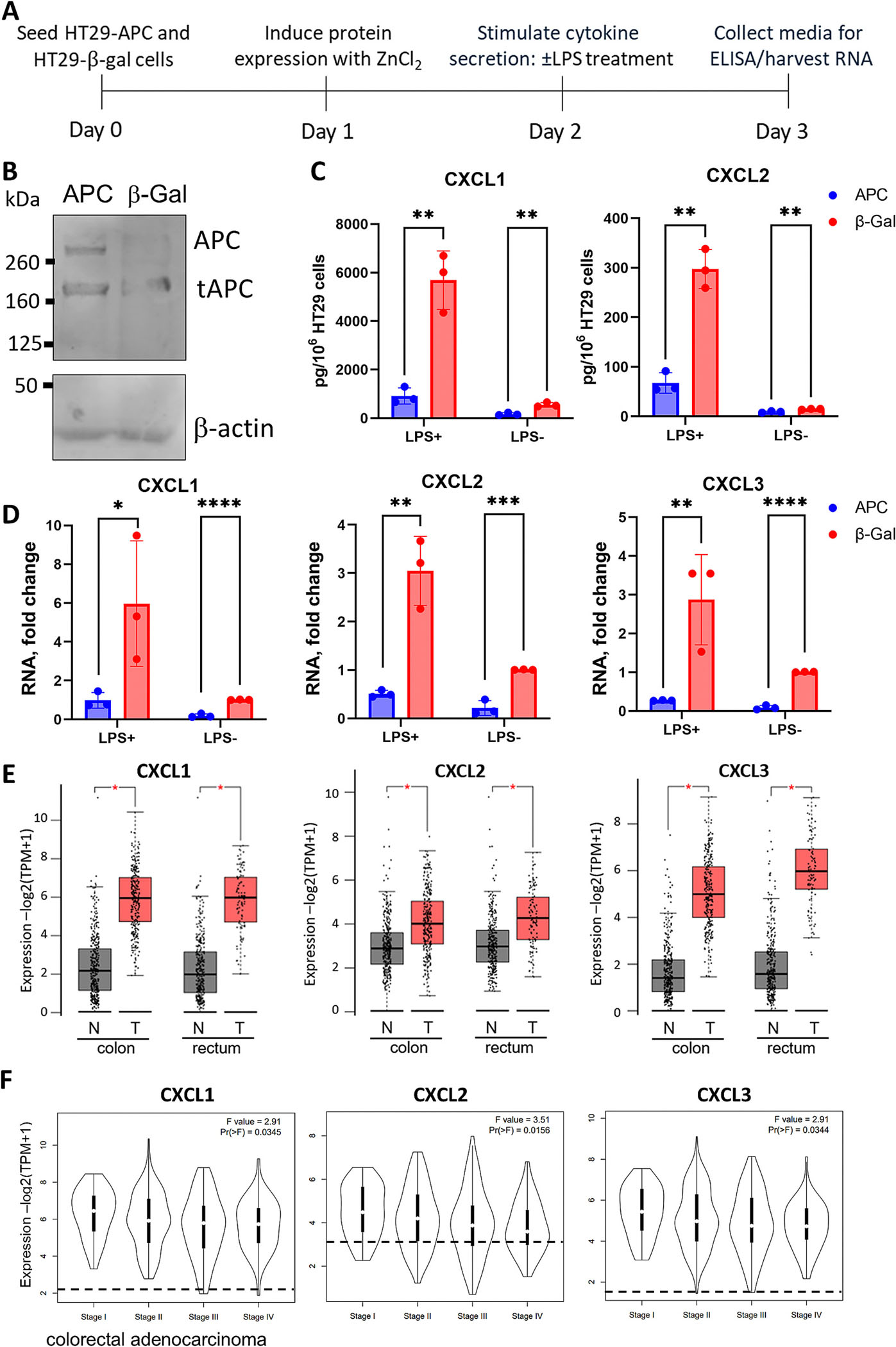
Induced APC expression in HT29 cells reduces CXCL1 and CXCL2 secretion and *CXCL1*, *CXCL2*, and *CXCL3* RNA levels. (A) Experimental outline. (B) Western blot confirming APC induction in HT29-APC, but not HT29-βGal cells following 48 h of ZnCl_2_ treatment. The band representing the largest of two endogenous truncated APC proteins (1555 amino acids) is marked as tAPC. (C) Graphs show levels of secreted CXCL1 and CXCL2 in conditioned media harvested from HT29-APC and HT29-β-gal cells. CXCL levels were standardized to cell count due to differing growth rates of the two cell lines (Supp. Fig. 1) and are presented as pg/10^6^ HT29 cells. APC expression significantly reduced CXCL1 and CXCL2 levels, both with and without added LPS. (D) *CXCL1*, *CXCL2*, and *CXCL3* RNA levels in zinc-induced HT29-APC and HT29-β-Gal cell lines, with and without LPS stimulation were normalized to levels from zinc-induced HT29-β-Gal cells without LPS (set to 1). Induction of APC decreased RNA levels of *CXCL1*–*3*. Data presented as mean ± SD from three independent experiments. Statistical significance was calculated using unpaired Student’s *t*-test. (E) Box Plots show RNA levels (−log2(TPM + 1)) for *CXCL1*–*3* in tumor (T, red) and normal (N, gray) tissues from colon and rectum as found in TCGA data using the GEPIA2 tool. TPM is Transcripts Per Kilobase Million. Colon samples included 275 T and 349 N; Rectal samples included 92 T and 318 N. Statistical significance was calculated using One-Way ANOVA. (F) Gene expression levels were assessed for CXCL1–3 and plotted for Pathological Stage using GEPIA2. Colorectal adenocarcinoma samples were grouped by stage. Dashed line indicates the average expression for normal colon (349) and rectal (318) tissues. F Value: The overall F-statistic of the ANOVA model; Pr(>F): The *p*-value associated with the F-statistic. (*P ≤ 0.05, **P ≤ 0.01, ***P ≤ 0.001,**** *P* ≤ 0.0001).

To investigate potential roles for APC in cytokine production and inflammation, we used the human colon cancer cell line HT29 which expresses endogenous truncated APC but can be induced with zinc to express full-length APC or β-galactosidase which serves as a negative control [33] (Fig. 1B, S1 A). LPS was optionally added to stimulate cytokine secretion (Fig. 1A). Induction of APC significantly reduced the amount of secreted CXCL1 and CXCL2 proteins measured in the media, regardless of LPS stimulation (Fig. 1C). This reduction of secreted CXCL1 and CXCL2 was more pronounced in APC-expressing cells also stimulated with LPS. Consistent with our previous finding that *CXCL2* (*MIP2*) RNA levels are elevated in colon epithelia from Apc^mNLS/mNLS^ mice [17], in the human colon cancer cells, APC expression markedly reduced levels of *CXCL2* RNA and also *CXCL1* and *CXCL3* RNA (Fig.1D). As further support that APC suppresses *CXCL* expression in humans, we found that the levels of *CXCL1, 2,* and *3* RNA were significantly higher in colon adenocarcinoma tissue than in normal colon tissue (Fig.1E). The same elevation of *CXCL 1, 2,* and *3* expression was observed in rectal adenocarcinoma compared to normal rectal tissue. Notably, these elevations were apparent in colorectal tumors as early as Stage 1, at which time *APC* mutations would already have been expected to occur (Fig. 1F, colorectal samples combined and S2, colon and rectal samples separated). *CXCL1, 2,* and *3* RNA elevation in early stages of colorectal cancer is consistent with a role for wild-type APC in inhibition of *CXCl1, 2*, and *3* expression in normal tissue.

### 3.2. APC increases RNA level of STAT1, a potential inhibitor of CXCL1 transcription

**Fig. 2.**
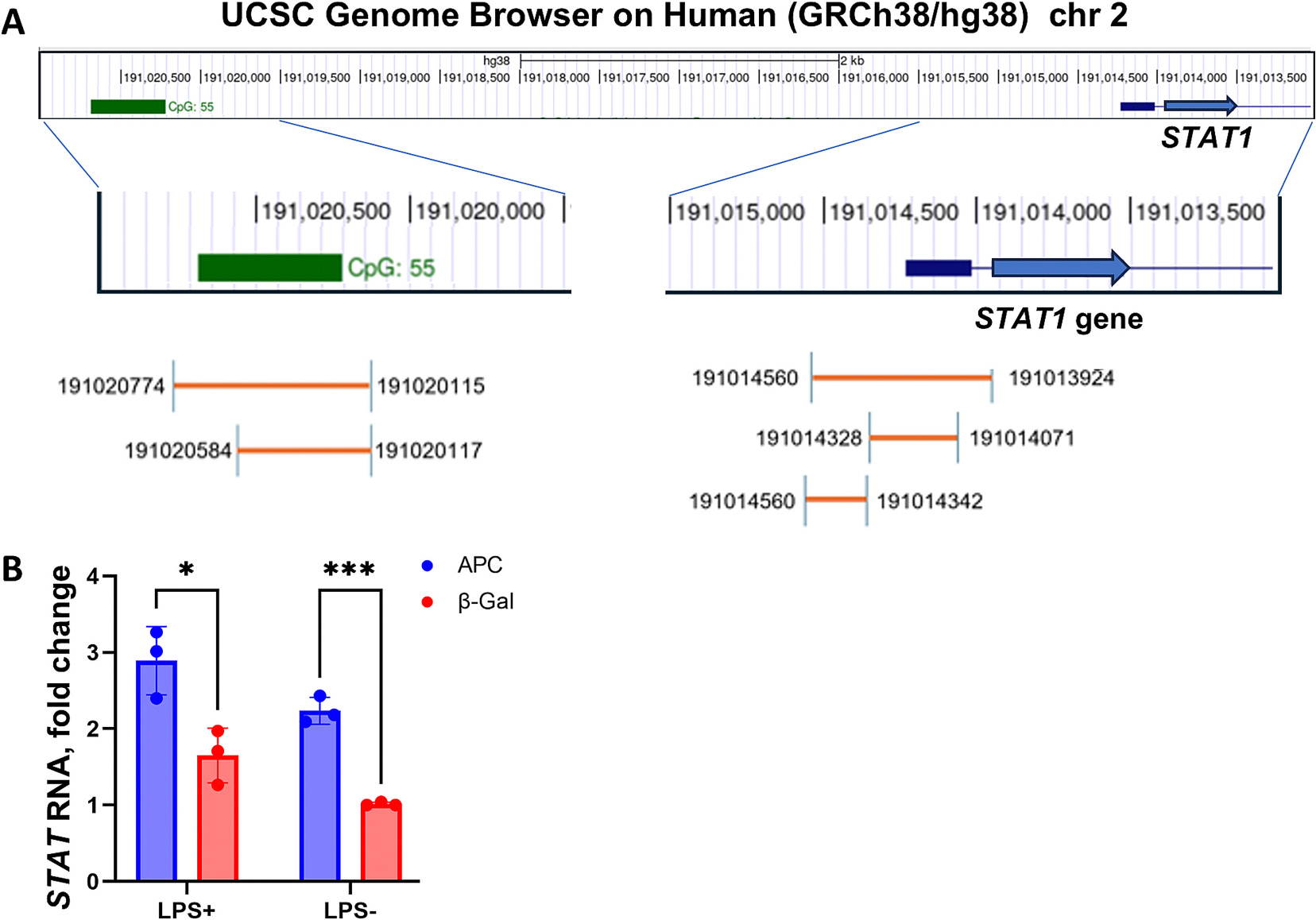
APC increases *STAT1* expression. (A) *STAT1* gene region of chr2 from UCSC Genome Browser on Human (GRCh38/hg38). Chromosome locations are shown above; scale bar = 2 kb. Below, expanded views of *STAT1* CpG island and promoter regions with fragments that we found associated with APC by mining a published ChIP-seq database from Hankey et al. [14]. APC-bound fragments are indicated as orange lines with chromosomal locations indicated. (B) Using the experimental design in Fig. 1A, APC expression in HT29 cells increased *STAT1* RNA level, regardless of LPS stimulation. Graph shows average fold-change of *STAT1* RNA in zinc-induced HT29-APC and HT29-β-Gal cell lines with and without LPS stimulation and normalized to levels from HT29-β-Gal cells without LPS (set to 1). Data presented as mean ± SD from three independent experiments. Statistical significance was calculated using unpaired Student’s *t*-test. (*P ≤ 0.05, **P ≤ 0.01, ***P ≤ 0.001).

To explore the mechanism by which APC down-regulates CXCL1–3, we first focused on the transcription factor STAT1. STAT1 can inhibit *CXCL1* expression by preventing binding of transcriptional activator Sp1 to the CXCL1 promoter [34–36]. Moreover, *STAT1* promoter sequences were included in published data sets from an APC ChIP-seq experiment performed by Hankey et al. using a human colon cell line [18]. Mining data from the APC ChIP-seq experiment, we found APC bound to 5 DNAs either in the promoter region or a CpG island about 5.5 kb upstream from the *STAT1* promoter (Fig.2A). Others have shown this CpG island to enhance STAT1 expression in luciferase reporter assays [37,38]. When we expressed APC in HT29 cells as in Fig. 1A, *STAT1* RNA levels increased, independent of LPS stimulation (Fig. 2B). This result supports the concept that in our cell system, APC promotes *STAT1* expression.

### 3.3. APC expression decreases methylation of STAT1 CpG island

**Fig. 3.**
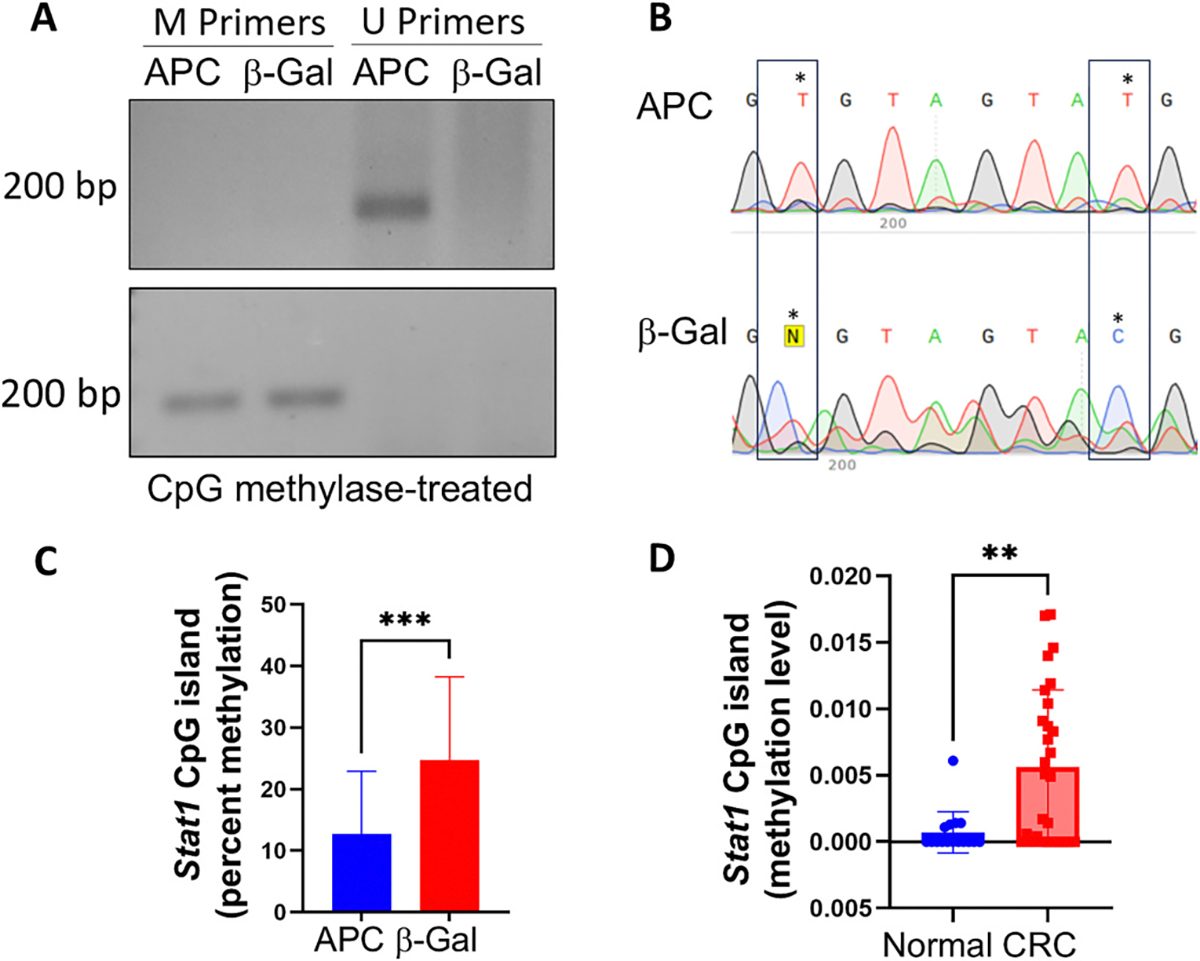
APC reduces methylation of *STAT1* CpG island. (A) (top) Primers that recognize Methylated (M) or Unmethylated (U) *STAT1* CpG island sequences were used to amplify sodium bisulfite-treated gDNA from HT29-APC and HT29-β-gal cells. Products resolved in 2 % agarose gel revealed no PCR amplification with M primers for either HT29-APC or HT29-β-gal gDNA. Using U primers, a band was amplified from HT29-APC but not HT29-β-gal gDNA, indicating that APC reduced methylation of the STAT1 CpG island. Gel shown is representative of three independent experiments. (bottom) gDNA from HT29-APC and HT29-β-gal cells was treated with methylase prior to bisulfite treatment and then PCR-amplified with methylation-specific (M) primers. Bands in each sample demonstrate the DNA fidelity and specificity of M primers. (B) Chromatogram showing sequence of two CG sites within the *STAT1* CpG island following bisulfite treatment of gDNA from HT29-APC or HT29-β-gal cells. Red thymine peaks and blue cytosine peaks clearly show a shift from C to T with APC expression. Loss of C peaks in the HT29-APC cells indicates no or only minimal methylation of the CpG island, whereas HT29-β-gal cells show partial CpG island methylation. (C) Average percent methylation for all *STAT1* CpG island CG sites combined as measured from sequencing bisulfite-treated HT29-APC and HT29-β-gal gDNA. HT29-APC cells showed significantly less *STAT1* CpG island methylation than HT29-β-gal cells. Data are expressed as means ± SD for 27 CG sites sequenced from three independent experiments. (D) Average methylation of the *STAT1* CpG island in colorectal cancer (*n* = 28) and normal (*n* = 16) colon tissue as determined using MethMarkerDB. Colorectal cancer tissue showed significantly higher methylation levels compared to normal tissue. Data presented as means ± SD. Statistical significance was determined using unpaired Student’s *t*-test (**P ≤ 0.01, ***P ≤ 0.001).

When methylated, CpG islands can reduce transcription of adjacent genes. Since cells induced to express APC showed higher *STAT1* RNA levels, we predicted that APC expression would result in less CpG methylation. To test this hypothesis, methylation-specific PCR (MSP) was performed on sodium bisulfite-treated genomic DNA (gDNA) from HT29-APC and HT29-β-gal cells (Fig. 3A, Supp. Fig. 3A). By treating the gDNA with sodium bisulfite, all unmethylated cytosine bases are converted to uracil. A set of primers specific for methylated (M) or unmethylated (U) DNA was used for PCR amplification to determine the methylation status of a specific segment of gDNA—in this case, a fragment of the *STAT1* CpG island. We found that no fragment was amplified using M primers from either HT29-APC or HT29-β-gal gDNA, suggesting methylation was incomplete if occurring at all. The M primers did amplify gDNA that was treated with DNA methylase which served as a positive control (Fig. 3A, bottom and Supp. Fig. 3A). Because the U primers amplified a CpG fragment from HT29-APC gDNA but not HT29-β-gal gDNA, we concluded that APC reduces methylation of the *STAT1* CpG island. To further confirm these results, we sequenced the *STAT1* CpG island in bisulfite-treated gDNA. Again, unmethylated cytosine bases are converted into uracil with sodium bisulfite and appear in the sequence as thymine bases. The average percentage of methylation at a given CG site was determined from the chromatogram using the cytosine peak height divided by the combined heights of cytosine and thymine [C/(C + T)]. Multiple cytosines in the CpG island showed higher cytosine and less thymine in the gDNA from HT29-β-gal cells than from the HT29-APC gDNA (Fig. 3B). This result was consistent with our previous observation of reduced methylation in the *STAT1* upstream CpG island in HT29-APC cells. On average, the CG sites of the CpG island were more methylated in HT29-β-gal cells compared to the HT29-APC cells (Fig. 3C). At the level of individual CG sites, the majority were less methylated in APC-expressing cells (Supp. Fig. 3B). To estimate the methylation level of the *STAT1* CpG island within human colorectal cancer patient samples, 80 % of which are expected to have mutant *APC,* we used the MethMarkerDB database. We found that the *STAT1* CpG island was more methylated in colorectal cancers than in normal colon tissue (Fig. 3D), consistent with our experimental data. Together, these data implicate APC in reducing methylation of the *STAT1* CpG island.

### 3.4. Mice with compromised nuclear import of Apc show increased ileal CXCL1 and CXCL2 production

**Fig. 4.**
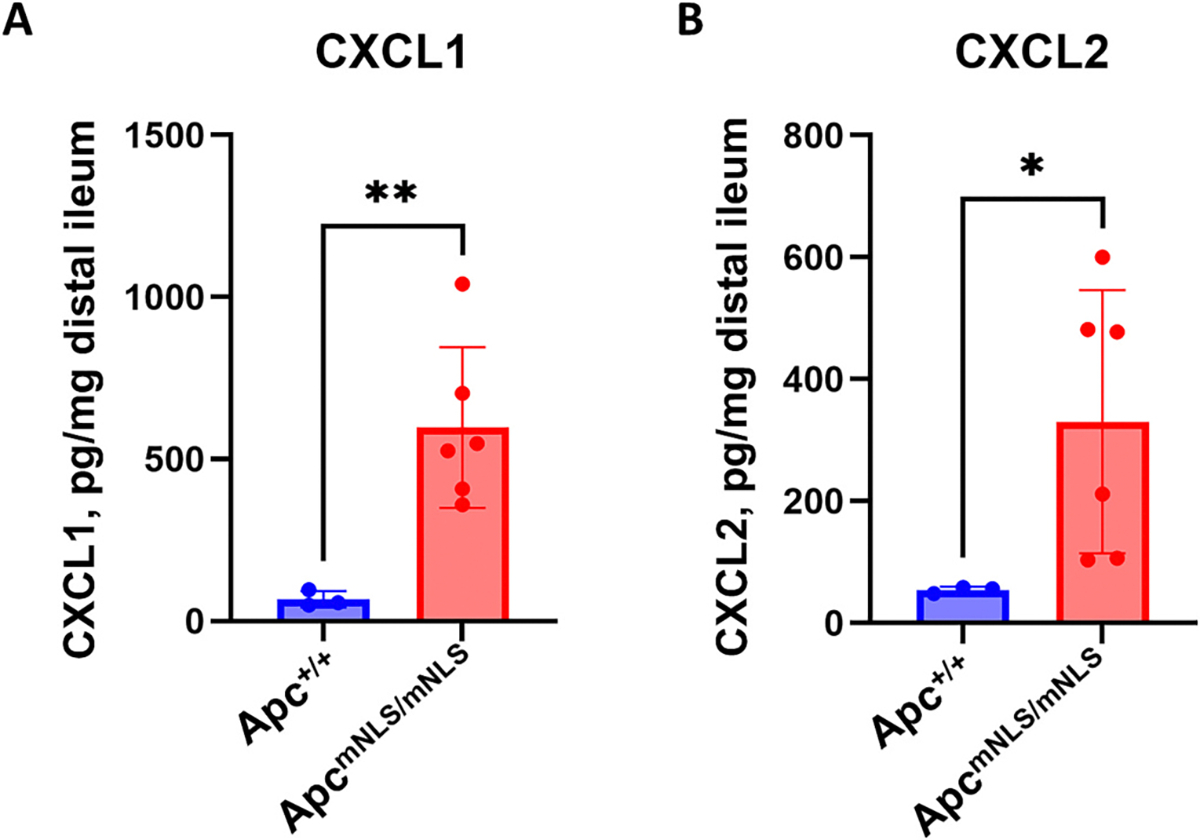
Ileal explants from Apc^mNLS/mNLS^ mice secrete more CXCL1 and CXCL2 than explants from Apc^+/+^ mice. Graphs show average level of CXCL1 (A, mouse KC) and CXCL2 (B, mouse Mip2) collected in the media from distal ileal explants of Apc^mNLS/mNLS^ and Apc^+/+^ mice. Secreted protein (pg) was standardized to explant mass. Explants from Apc^mNLS/mNLS^ mice secreted significantly more CXCL1 and CXCL2 than the explants from Apc^+/+^ mice. Data presented as means ± SD for 3 Apc^+/+^ and 6 Apc^mNLS/mNLS^ explants. Statistical significance was calculated using unpaired Student’s t-test (*P ≤ 0.05, **P ≤ 0.01).

After identifying APC as an inhibitor of *CXCL1*–*3* and inducer of *STAT1* expression in vitro, as well as a linking APC with decreased methylation of *STAT1* promoter-adjacent CpG islands, we turned to an in vivo model to explore nuclear APC functions. Distal ileal tissues were excised from mice with compromised nuclear Apc import (Apc^mNLS/mNLS^) and incubated in RPMI media. The conditioned media was then analyzed for CXCL1 and CXCL2 (KC and MIP-2 in mice) protein concentration via ELISA (Fig. 4A and B). Media from Apc^mNLS/mNLS^ explants contained significantly more CXCL1 and CXCL2 compared to media from Apc^+/+^ explants. These results using animal tissue are consistent with our observations that APC inhibited CXCL1 and 2 in vitro. They also expand our understanding by implicating specifically nuclear APC in CXCL1 and 2 inhibition.

### 3.5. APC inhibits neutrophil migration

**Fig. 5.**
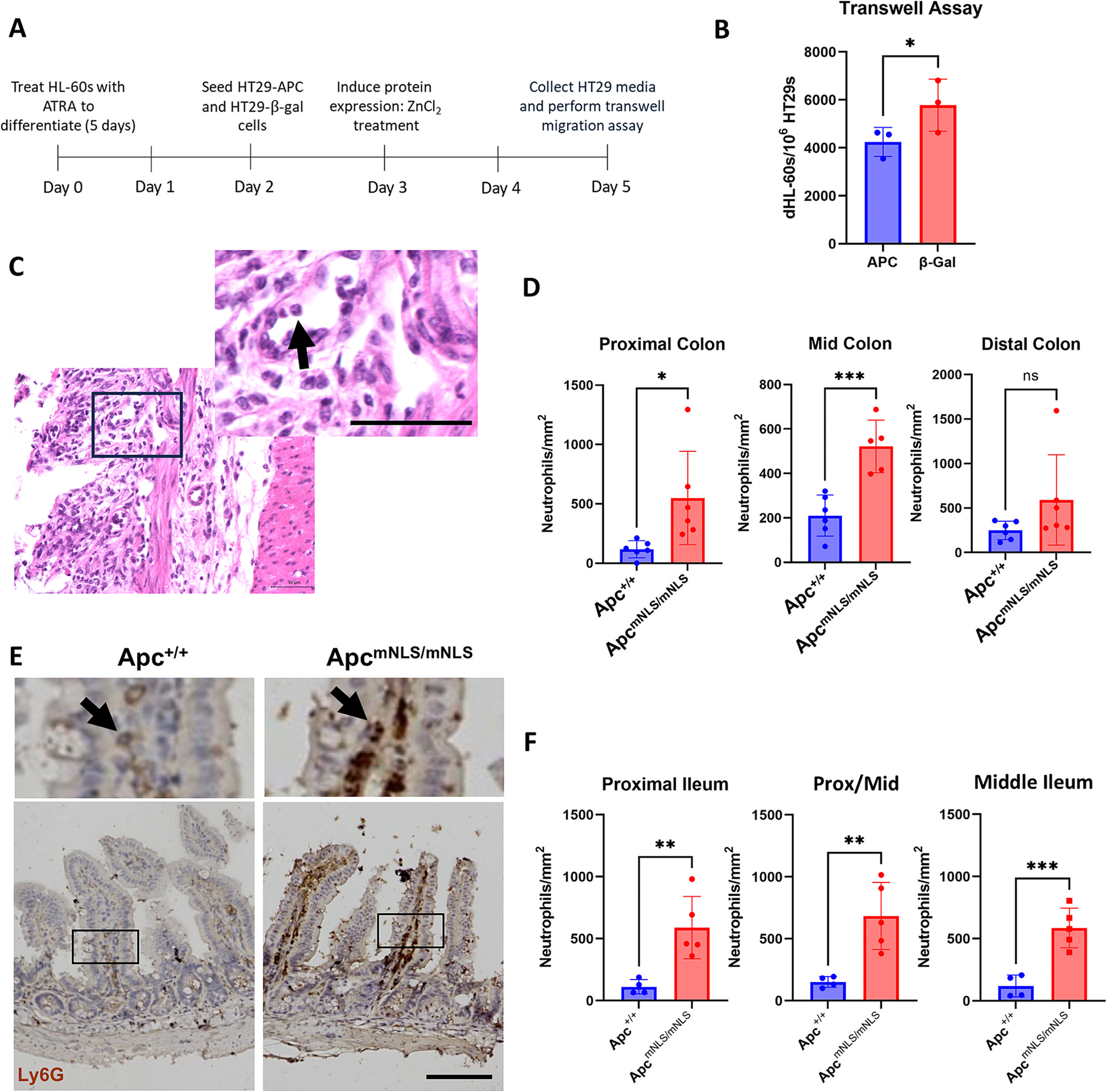
APC inhibits neutrophil migration. (A) Experimental outline. (B) Graph shows average number of neutrophil-like dHL-60 cells that migrated through pores of transwell inserts into conditioned media harvested from HT29-APC and HT29-β-gal cells. Neutrophil counts were standardized to the number of HT29 cells from which the media were isolated. Significantly fewer dHL-60s migrated through the inserts when HT29-APC-conditioned media was used compared to when HT29-β-gal conditioned media was used. Data are shown as means ± SD of three independent experiments. Statistical significance was calculated using paired Student’s t-test (*P ≤ 0.05). (C) Neutrophils in H&*E*-stained colon tissue sections from DSS-treated Apc^mNLS/mNLS^ and Apc^+/+^ mice were identified based on their multi-lobed nuclear morphology. Figure shows an example of a neutrophil in the proximal colon of an Apc^mNLS/mNLS^ mouse. Photo was taken at 40× magnification. Scale bar 50 μm (D) Apc^mNLS/mNLS^ mice had significantly more neutrophils per area than Apc^+/+^ mice in the proximal and mid colon and a trend (*P* = 0.0685) in the same direction for neutrophils in the distal colon. Data are expressed as means ± SD; *n* = 6 Apc^+/+^ mice, n = 6 Apc^mNLS/mNLS^ mice. (E) Neutrophils in ileal tissue were identified by Ly6G stain and scored as in C. Scale bar 100 μm. (F) Apc^mNLS/mNLS^ mice had significantly more neutrophils per area than Apc^+/+^ mice in all ileal sections. Data are expressed as means ± SD; *n* = 4 Apc^+/+^ mice, *n* = 5 Apc^mNLS/mNLS^ mice. Statistical significance was calculated using unpaired Student’s t-test (*P ≤ 0.05, ***P ≤ 0.001).

Having established that APC suppresses *CXCL1, 2,* and *3*, enhances *STAT1* expression, and reduces *STAT1* CpG island methylation, we turned to the potential consequences of APC chemokine suppression. CXCL1, CXCL2, and CXCL3 are well-established promoters of neutrophil recruitment. We used a transwell migration assay [39] to assess the migration of neutrophil-like differentiated HL-60 cells (dHL-60) toward conditioned media collected from HT29-APC and HT29-β-gal cells (Fig. 5A). We found that fewer dHL-60 cells migrated into conditioned media from HT29-APC cells than into media from HT29-β-gal cells (Fig. 5B). If wild-type APC represses expression of CXCL1–3 in vitro, and tissue explants from Apc^mNLS/mNLS^ mice with compromised nuclear import of APC produce and secrete significantly more CXCL-1 and -2 (Fig. 4), we would expect to observe more neutrophils in the tissues from these mice. Neutrophils were assessed in the proximal, mid, and distal colons of Apc^mNLS/mNLS^ mice treated briefly with DSS to induce colitis. Compared to wild-type, Apc^+/+^ mice, we found that Apc^mNLS/mNLS^ mice had significantly more neutrophils in the lamina propria of the proximal and mid colon (Fig. 5C and D). The distal colon also showed a trend of more neutrophils in Apc^mNLS/mNLS^ mice compared to Apc^+/+^ mice (*P* = 0.0685). We observed a similar increase in neutrophil numbers in ileal tissue from untreated Apc^mNLS/mNLS^ mice compared to Apc^+/+^ mice (Fig. 5E and F). Thus, we established that, compared to their wild-type littermates, Apc^mNLS/mNLS^ mice both secrete more CXCL1 and 2 in their ilea and have more neutrophil infiltration.

## 4. Discussion

Inflammation is now recognized as a tumor-promoting characteristic that enables cancer cells to acquire the other ten hallmark capabilities, shared by most tumors [40,41]. The role of inflammation in carcinogenesis is complex, with inflammatory signals both protecting against and promoting cancer. Further complexity arises when we consider the role of inflammation in different tissues and the interplay of inflammation and key driver mutations in various tumor suppressors and oncogenes. Though colon epithelial tissue is particularly susceptible to tumor initiation by APC inactivation and chronic inflammation is a risk factor for CRC, the role of APC in inflammation is not well-defined. To our knowledge, our study provides the first evidence for a direct connection between APC, *CXCL* and *STAT1* expression, and neutrophil attraction.

To further understand APC’s role in inflammation, we first investigated whether APC regulates neutrophil-recruiting chemokines CXCL1, 2, and 3. We found that induction of functional, full-length APC in human colon cancer cells reduced the levels of secreted neutrophil-recruiting chemokines CXCL1 and CXCL2, accompanied by a reduction in neutrophil recruitment. Moreover, human cancer tissues from both colon and rectum express significantly more *CXCL1*–*3* than normal tissue. The vast majority of these cancers are expected to have inactivated APC as an early step in their progression. Notably, the increased levels of *CXCL 1*–*3* expression are already observed in Stage 1 tumors and, in the case of CXCL2, decline to near normal levels at later cancer stages (Fig. 1F and supp. Fig. 2). This fluctuation of CXCL expression might reflect different roles for these cytokines at different stages of tumorigenesis.

We previously showed that a subset of goblet cells displayed elevated levels of APC and that these APC^high^ goblet cells increased in number in mice with induced colitis or in colons of UC patients [31]. At face value, this observation of higher APC levels in cells undergoing inflammation seems inconsistent with our current results which implicate APC in an anti-inflammatory process. This discrepancy could be rationalized if APC upregulation in the setting of colitis is part of a feedback response to resolve the inflammatory response. Alternatively, APC could have disparate roles in inflammation, depending on the context. Other published data are consistent with our observation of elevated CXCL1–3 expression at early stages of carcinogenesis and support an anti-inflammatory role for APC. Polyps from Apc^Min/+^ mice display increased expression of inflammatory mediators, including *CXCL1* and *CXCL2* [42]. Small intestine organoids from Apc^null^ mice also displayed significant elevation of *CXCL1* and *CXCL2* RNA [43]. Familial adenomatous polyposis (FAP) patients (germline *APC* mutation) showed elevated levels of both *CXCL2* and *3* in polyps vs. normal tissue [44]. In contrast, APC was reported to upregulate CXCL2 by forming a complex with transcription factor C/EBPβ in macrophages exposed to *Bacillus anthracis* edema toxin [45]. Though we provide evidence that APC can reduce chemokine production in colon epithelium, APC effect on chemokines is likely cell- and context-dependent.

Based on previous evidence that STAT1 can inhibit *CXCL1* expression in certain cell lines [34–36] we hypothesized that APC indirectly inhibits CXCL1–3 by promoting *STAT1* expression. We mined previously published APC ChIP-seq data [18] and identified an interaction between APC and DNAs corresponding to the *STAT1* promoter and upstream CpG island. We confirmed that APC expression leads to increased *STAT1* RNA. APC and STAT1 connections have been reported previously. Organoids from attenuated FAP adenomas generally expressed less *STAT1* RNA than organoids from classic FAP cases [46]. This result contrasts with our findings that APC can increase *STAT1* expression in HT29 colon cancer cells and that *STAT1* CpG island is more methylated in human CRC when compared with normal tissue. One obvious difference in these systems is that the FAP study used adenoma tissue, while ours focused on a cancer cell line or colorectal cancer patient samples. The additional gene alterations expected to accompany transitions from adenoma to carcinoma no doubt also impact *STAT1* expression. Moreover, with the many established regulatory mechanisms for *STAT1* expression, it is not surprising that different results are observed using diverse models, tissues, and states of carcinogenesis. Finally, a possible negative feedback loop whereby STAT1 can inhibit *APC* transcription adds to the complexity [47].

We also showed that APC induction reduces methylation of the endogenous *STAT1* CpG island. Taken together, our results suggest that APC increases *STAT1* expression by recruiting transcription factors to the promoter, by reducing methylation of the *STAT1* CpG island, or by both mechanisms. Methylation-specific PCR and bisulfite sequencing results indicated that APC reduced methylation of the endogenous *STAT1* CpG island. Whereas APC has been shown to bind to DNA [48], to our knowledge, ours is the first study to suggest that nuclear APC regulates transcription via altering the methylation status of CpG islands. We speculate that APC may bind to the CpG island directly or indirectly, physically blocking the DNA methyltransferases from adding methyl groups. Another potential mechanism for APC to alter *STAT1* and *CXCL1*–*3* expression is through its role as a Wnt signal antagonist. In this capacity, we would expect APC to directly block β-catenin binding to *STAT1* and *CXCL1*–*3* DNA in the nucleus or indirectly through cytoplasmic β-catenin degradation. Because there is no evidence for β-catenin interaction with *STAT1* or *CXCL1*–*3* DNAs in a ChIP-seq experiment [49] which used the same cell lines as the APC ChIP-seq study [18], we think that this possible mechanism is less likely.

Our primary goal in this study was to determine mechanism(s) underlying nuclear APC’s anti-inflammatory phenotype that we had previously observed in Apc^mNLS/mNLS^ mice. To gain mechanistic insight, we moved into an in vitro system, with accompanying limitations. The HT29-APC cells were induced to express full-length, wild-type APC, capable of localizing to both the cytoplasm and nucleus and thus making it difficult to distinguish nuclear-specific properties. To overcome this limitation, after testing different mechanisms using the in vitro system, we returned to the Apc^mNLS/mNLS^ mice. Using this in vivo model, we showed increased CXCL1 and CXCL2 secretion from ileal tissue explants and increased neutrophil infiltration in both colon and ileal tissue from mice with compromised nuclear Apc. To our knowledge, the current report is the first to demonstrate a role for nuclear APC in regulating CXCL1 and CXCL2 and subsequent neutrophil chemoattraction in intestinal tissue. We show that APC can suppress *CXCL1*, *CXCL2*, and *CXCL3* and promote *STAT1* expression and provide evidence that APC can act by reducing methylation of the *STAT1* CpG island. We previously reported that Apc^mNLS/mNLS^ mice are prone to colitis and that inflamed colons of both mice and humans display elevated APC in a subset of goblet cells [17,31]. The results from the current study provide evidence that nuclear APC can inhibit inflammation. APC loss in early stages of colorectal carcinogenesis would therefore be expected to promote an inflammatory signal and thus enable downstream characteristics necessary for cancer progression.

## Supplementary Material

Supplemental Figure 1

Supplemental Figure 2

Supplemental Figure 3

Supplementary data to this article can be found online at https://doi.org/10.1016/j.cellsig.2025.111957.

## Figures and Tables

**Table 1:** Human primer sequences for qRT-PCR.

Gene	Forward primer	Reverse primer

*CXCL1*	ATTCACCCCAAGAACATCCA	CACCAGTGAGCTTCCTCCTC
*CXCL2*	GCAGGGAATTCACCTCAAGC	AGCTTCCTCCTTCCTTCTGG
*CXCL3*	GCAGGGAATTCACCTCAAGA	GGTGCTCCCCTTGTTCAGTA
*STAT1*	TGTATGCCATCCTCGAGAGC	AGACATCCTGCCACCTTGTG
*GAPDH*	AGGTCGGTGTGAACGGATTTG	TGTAGACCATGTAGTTGAGGTCA

**Table 2:** Human *STAT1* promoter CpG island primer sequences for MSP.

Methylation status	Forward primer	Reverse primer

Methylated	CGTTTCGGAAATAGTTGGTTC	GCCTACGTACTACGCCTACGTA
Unmethylated	AATTGTTTTGGAAATAGTTGGTTTG	CTACACCTACATACTACACCTACATA

**Table 3:** Human *STAT1* CpG island primers for PCR amplification and bisulfite sequencing.

Primer	Sequence

Forward	GAAGGATTGGGATTGAGAGGAAA
Reverse	CCTCCCTCCCTCTAAAATAAAAC

## Data Availability

Data will be made available on request.

## Abbreviations:

APC: Adenomatous Polyposis Coli
IBD: Inflammatory Bowel Disease
UC: Ulcerative Colitis
CRC: colorectal cancer
DSS: Dextran Sodium Sulfate
FAP: Familial Adenomatous Polyposis
COX-2: Cyclooxygenase-2
MIP-2: macrophage inflammatory protein-2
CXCL1, 2, and 3: C-X-C motif chemokine ligand 1, 2, and 3
ChIP-Seq: Chromatin-immunoprecipitation sequencing
LPS: Lipopolysaccharide
STAT1: signal transducer and activator of transcription 1
MSP: methylation-specific PCR
RLU: relative light units
