# Supplementary figures and images for: Nuclear adenomatous polyposis coli elevates STAT1 and reduces CXCL1,2, and 3 expression and inhibits neutrophil recruitment
Source: Cell Signal. Author manuscript; Available in PMC 2026 Jun 1. (PMC13224742; doi:10.1016/j.cellsig.2025.111957)

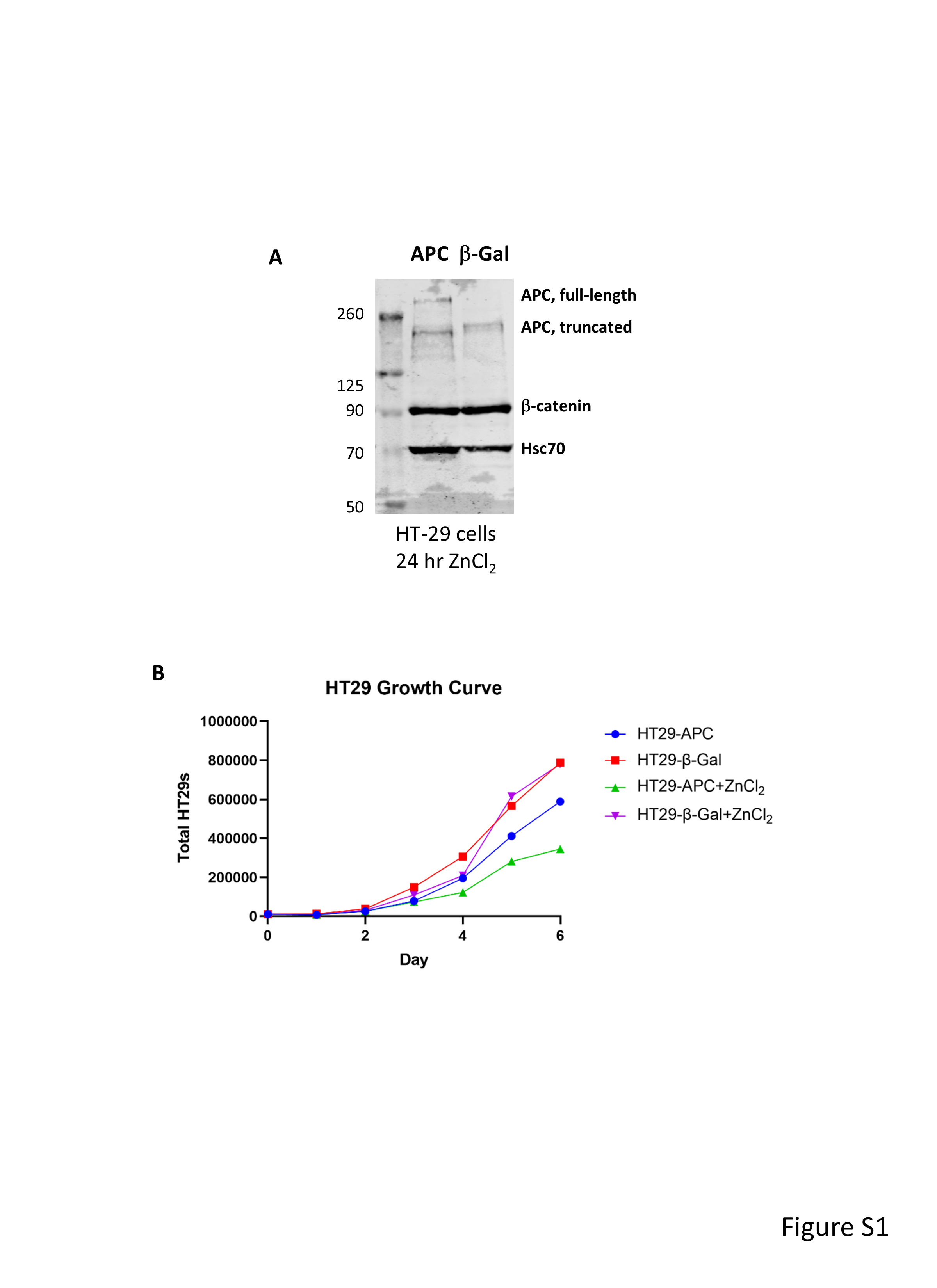

Supplement: Supplemental Figure 1 [file NIHMS2170674-supplement-Supplemental_Figure_1.tif]

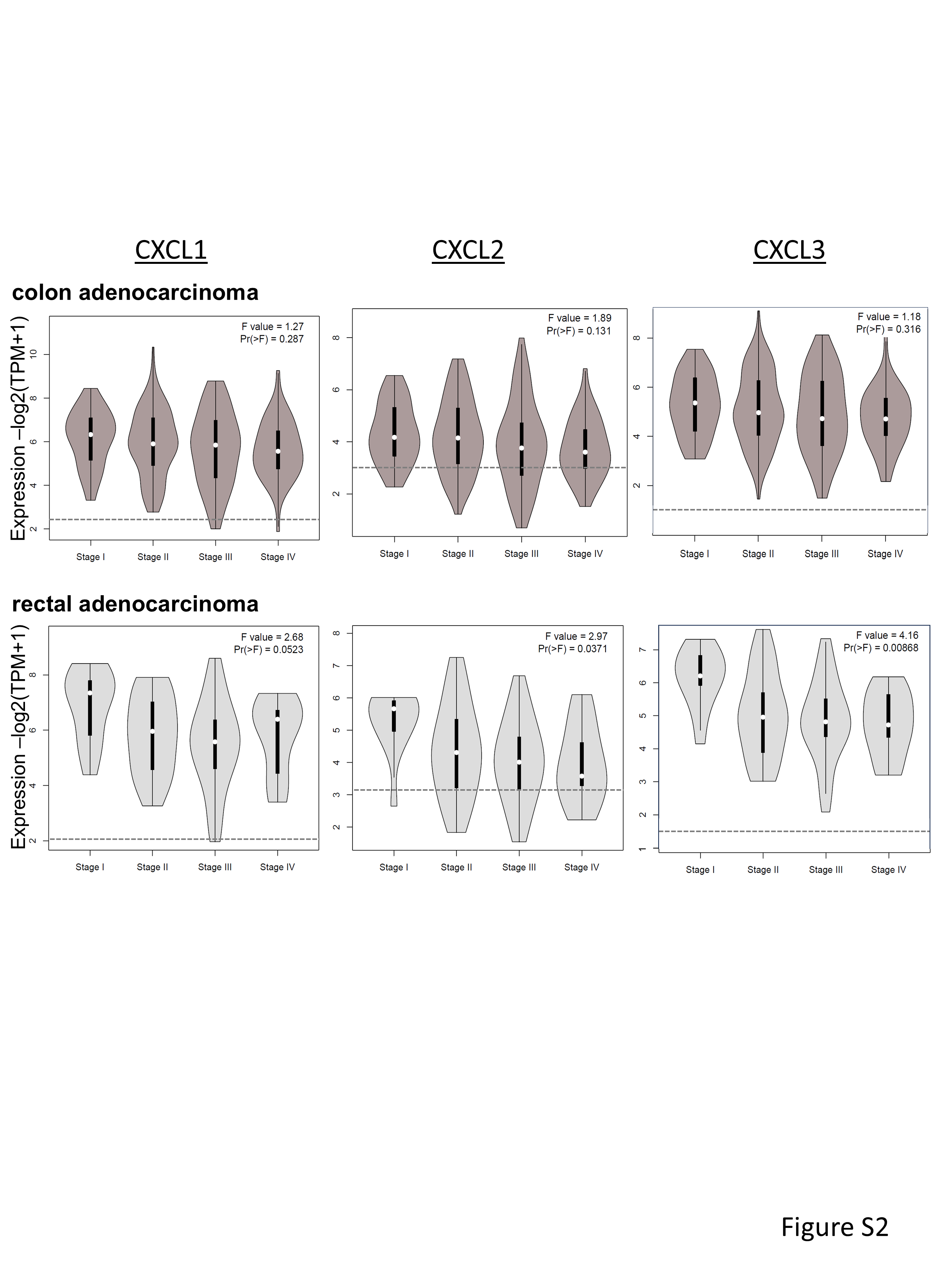

Supplement: Supplemental Figure 2 [file NIHMS2170674-supplement-Supplemental_Figure_2.tif]

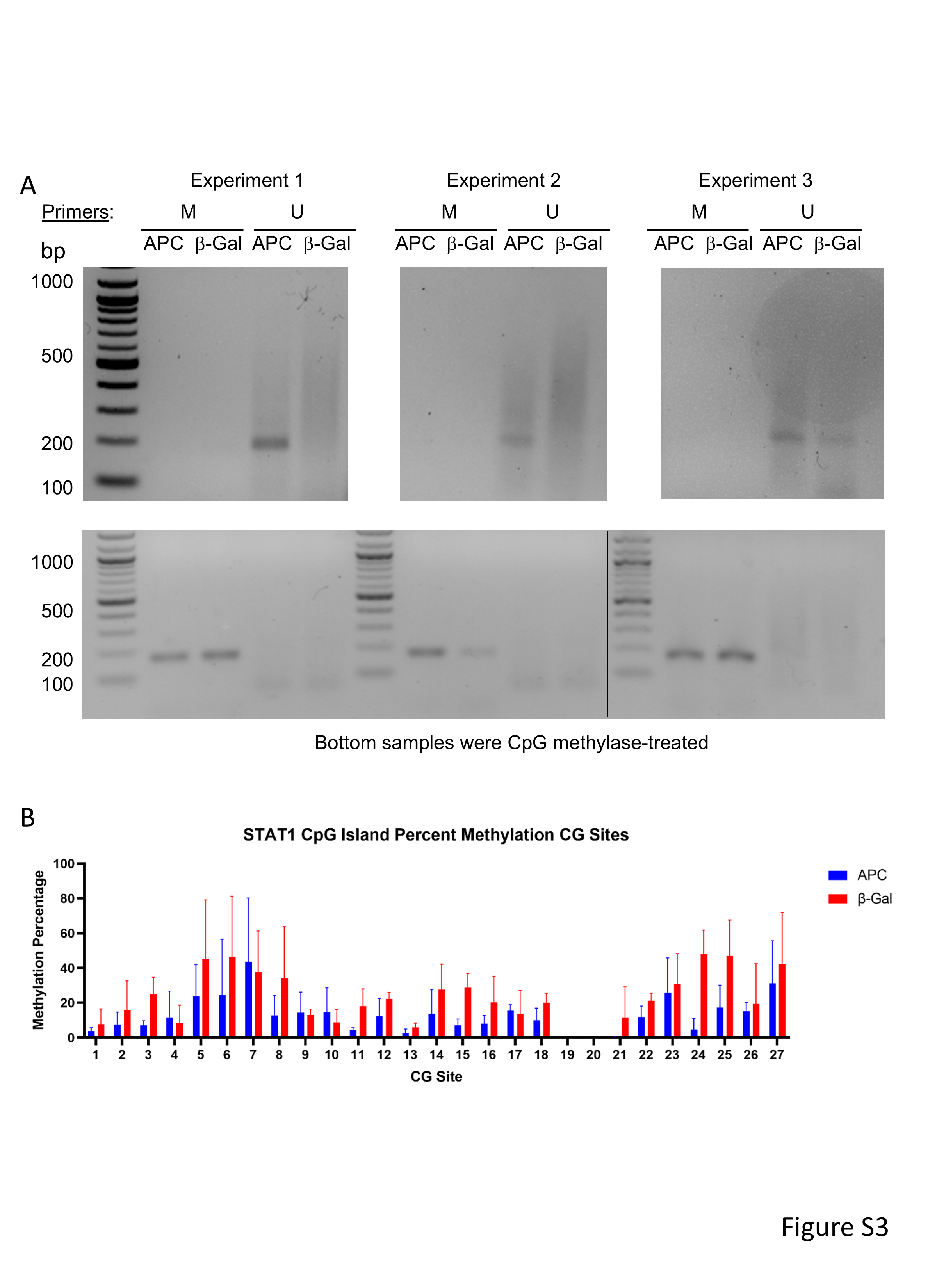

Supplement: Supplemental Figure 3 [file NIHMS2170674-supplement-Supplemental_Figure_3.tif]
